# Biopsychosocial predictive factors for developing chronic postsurgical pain after hip replacement surgery: A systematic review

**DOI:** 10.1016/j.ocarto.2025.100725

**Published:** 2025-12-04

**Authors:** Rachel J.H. Smits, Rosa Poppen, Jetze Visser, Kris C.P. Vissers, Selina E.I. van der Wal

**Affiliations:** a Department of Anesthesiology, Pain- and Palliative Medicine, Radboudumc, 6500 HB, Nijmegen, the Netherlands; b Department of Orthopedic Surgery, Radboudumc, 6500 HB Nijmegen, the Netherlands

**Keywords:** Chronic pain, Prehabilitation, Total hip arthroplasty, Chronic postsurgical pain, Anesthesiology

## Abstract

**Objective:**

Total hip arthroplasty for end stage osteoarthritis is a common and often successful procedure. However, 14 ​% of patients experience chronic postsurgical pain after 1 year. The risk of developing chronic pain is multifactorial. The aim of this systematic review is to provide an overview of recent literature on predictive factors for developing postsurgical chronic pain after total hip arthroplasty to create a prognostic model for better recognition, perioperative optimization or reconsideration.

**Design:**

Studies were eligible if patients were over 18 years old, biopsychosocial risk factors and the occurrence of pain >3 months after surgery was reported. PubMed, EMBASE and CENTRAL were searched up to June 16th, 2025. The selected studies were screened and assessed on quality and risk of bias.

**Results:**

Fifteen studies met the inclusion criteria. These studies identified multiple biopsychosocial factors that may contribute to the development of chronic postsurgical pain. These include body mass index, preoperative (neuropathic) pain, the presence of comorbidities and functional disability, smoking, non-white race, sleep disturbances, depressive symptoms, anxiety, central sensitization, and pain catastrophizing. Additional associations were found for (younger) age and female sex. Some studies failed to demonstrate significant associations of certain factors, highlighting the complexity of chronic pain.

**Conclusion:**

The identified, modifiable risk factors could be targets for prehabilitation and optimization. Personalized multimodal analgesia strategies might be more beneficial in high risk patients. Chronic postsurgical pain should be part of the informed consent and non-operative options could be considered in high risk patients.

## Graphical abstract

**Figure:**
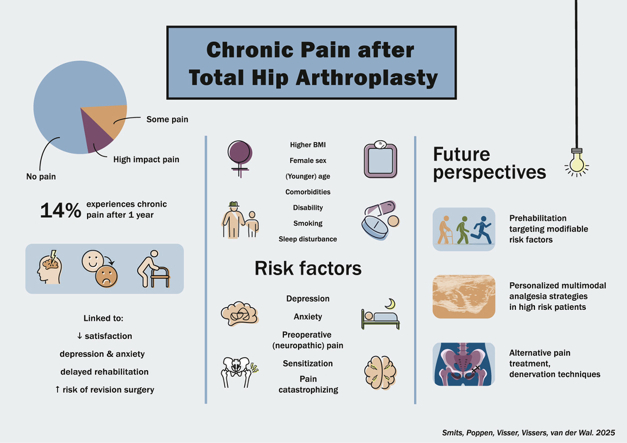

## 1 Introduction

The number of total hip arthroplasties is rising worldwide, improving mobility and reducing pain, yet 14 ​% of the patients experiences chronic postsurgical pain (CPSP) after 1 year [1,2]. This incidence is not decreasing despite the advancements in surgical technique and perioperative care [3]. CPSP lowers the quality of life, and is linked to depression, anxiety, and other psychological issues [4]. It can delay rehabilitation, reduce satisfaction, and increase the risk of revision surgery. The increased healthcare utilization and work absence imposes significant economic burden [5,6].

The pathophysiological mechanisms of CPSP after THA is unclear. In most cases, it is not directly attributable to nerve injury. Multiple factors influence the pain intensity and duration [[7], [8], [9]]. Risk factors include non-modifiable (such as age, sex, race, socio-economic background) or modifiable ones (including mental health status, sleep quality, obesity, smoking [10]. Since the last published systematic review, multiple studies have been published on CPSP after THA, highlighting the importance of the subject.

The objective of this review is to provide a comprehensive overview of the biological, psychological, and socio-economic risk factors that contribute to CPSP after THA. It aims to improve identification of high-risk patients, targeted prehabilitation, optimizing multimodal approach, and better informed-consent in these patients [11].

## 2 Materials and methods

The systematic review was performed following the Preferred Reporting Items for the Systematic Reviews and Meta-Analysis (PRISMA) 2020 guidelines [12] and was registered at January 11th^,^ 2023 in PROSPERO (CRD42023390761, www.crd.york.ac.uk/prospero/display_record.php?RecordID=390761). The protocol has not been published elsewhere.

### 2.1 Study eligibility

Eligible studies enrolled adults (≥18 years old) undergoing primary THA for any diagnosis. If other joint arthroplasties were included, THA data had to be reported separately. Eligible studies assessed factors influencing persistent pain ≥3 months after surgery. Definitions of pain scores varied and are detailed in the results and supplementary tables. All preoperatively assessed patient-related biopsychosocial and economic predictors were included. Randomized controlled trials and longitudinal cohort studies, both retrospective and prospective, were eligible. Case reports, abstracts without full publications, and non-English publications were excluded. Studies published before 2010 were excluded, to reflect the current perioperative standards.

### 2.2 Search strategy

The search strategy was based on a PICO-formulated question with assistance from a medical library specialist. The search combined population terms (e.g. ‘hip arthroplasty’, ‘hip replacement surgery’) with outcome terms (e.g. ‘chronic pain’, ‘persistent pain’, ‘postoperative pain’). PubMed, EMBASE, and the CENTRAL were searched on December 6th^,^ 2022, and updated on 27th 2024 and June 16th^,^ 2025. Full search strategies are available in supplement A.

### 2.3 Data extraction

The selected articles were exported to EndNote20, duplicates were removed electronically, and imported in Rayyan [13]. One reviewer (RP) screened titles and abstracts, excluding clearly irrelevant articles, and marking others as ‘possibly eligible’. A second, non-blinded, reviewer (RS) finalized the selection in Rayyan. Full texts of these articles were retrieved, screened, and assessed for eligibility and relevance by both reviewers independently (RP and RS). Data extracted included country, study design (e.g., inclusion and exclusion criteria, inclusion period and follow-up), patient demographics, predictors, outcome measures, statistical methods, and results. Primary outcome was the presence of pain at least 3 months post-surgery. Patient reported pain severity depended on each study's method.

### 2.4 Quality assessment

Risk of bias assessment was performed by two reviewers (RP and RS) and reviewed by another (KV) using the Quality in Prognostic Studies, as recommended by the Cochrane Prognosis Methods Group. Quality in Prognostic Studies covers study participation, attrition, prognostic factor measurement, outcome measurement, confounding, and statistical analysis [14]. Each item was rated low, moderate, or high risk of bias. Moderate or high risk of bias was defined as:

• Study participation: missing inclusion or exclusion criteria
• Study attrition: follow-up below 80 ​%
• Study confounding: no confounders mentioned

The quality of the evidence was rated by using the Grading of Recommendations Assessment, Development and Evaluation (GRADE) approach as ‘High’, ‘Moderate, ‘Low’ and ‘Very Low’ [15,16].

### 2.5 Data synthesis

A qualitative descriptive analysis was performed following the PRISMA statement. A meta-analysis was not conducted due to the substantial heterogeneity in outcome and the limited number of studies per category.

## 3 Results

### 3.1 Study selection and characteristics

**Fig. 1:**
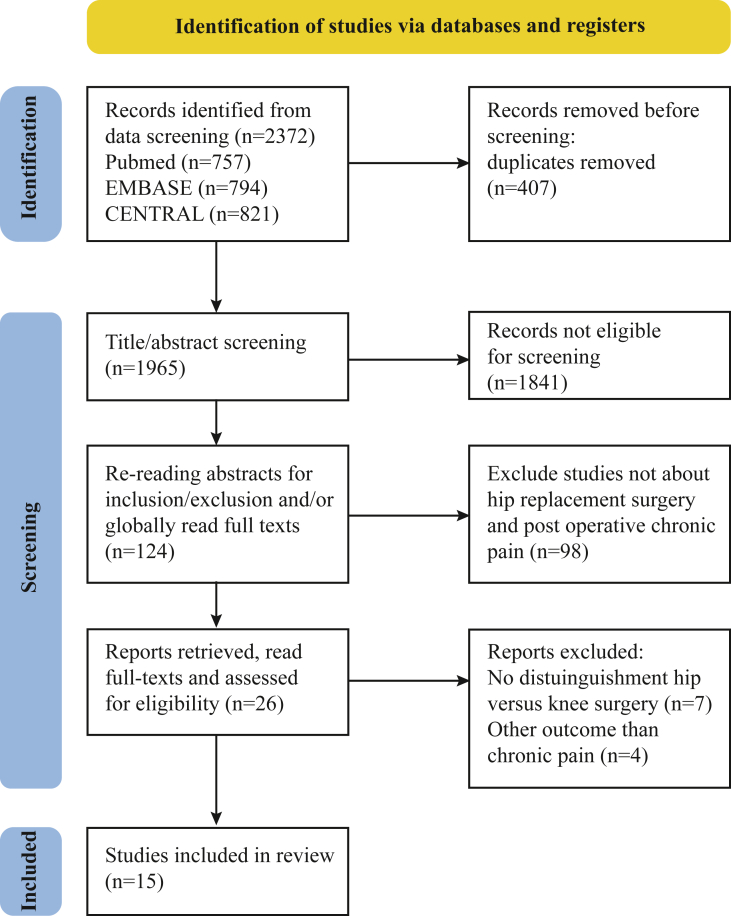
PRISMA flow diagram.

After removing duplicates, 1965 articles were screened. After the initial screening (RP), 124 potentially relevant articles were identified, and title and abstract were screened by two reviewers (RP and RS). The full texts of twenty-six articles were retrieved for full-text screening by both reviewers, which left fifteen eligible studies (Fig. 1). The most common exclusion criteria were the lack of specified results for THA or a different outcome. Table 1 and Supplement B summarize study characteristics and results. The results include 13 cohort studies, one pooled-cohort analysis (Hofstede et al. [17]) and one cohort analysis of two randomized controlled trials (Wylde et al. [18]). Eight studies also included total knee replacement surgery and two included total shoulder arthroplasty. Three studies did not separate demographic data, but presented their final results separately for THA patients or adjusted for the technique via regression analysis (George et al. [19], Paredes et al. [20], Boye Larsen et al. [21]). Pain assessments varied, using outpatient visits, postal questionnaires, or telephone interviews. One study did not specify the method of data collection.

**Table 1:** Summary of study characteristics and results.

	Design	N ​=	Follow up	Included factors	Outcome	Results
Bjurström 2021	Prospective cohort	52	6 months	Pittsburgh Sleep Quality Index (PSQI), brief pain inventory short form (BPI-sf), douleur neuropathique 4 (DN4), pain catastrophizing scale (PCS), western Ontario and McMaster universities osteoarthritis index (WOMAC), hospital anxiety and depression scale (HADS)	BPI-SF (0-10), WOMAC	Preoperative sleep disturbances was associated with higher pain severity 6 months after THA (adjusted for demographic factors and HADS score)
Blikman 2024	Prospective cohort	245	12 months	Age, sex, BMI, preoperative pain intensity (NRS 0–10) and duration (months), neuropathic-like symptoms via the modified painDETECT questionnaire (mPDQ)	Oxford hip score (OHS), NRS (0–10)	Preoperative neuropathic-like pain was a predictor for unfavorable long-term pain outcome
Boye larsen 2021	Retrospective cohort	89	12 months	PSQI, PCS, HADS, pain in hip (VAS 0–10)	VAS (1-10)	Preoperative pain catastrophizing, anxiety, depression and reduced sleep quality were no independent risk factors for chronic pain postoperatively
Erlenwein 2017	Prospective cohort	104	6 months	Age, sex, BMI, pain history (German pain questionnaire), preoperative medication use (MQS), severity of chronic pain (CPG), chronicity of patients condition (MPSS), preoperative hip pain (NRS 0–11), neuropathic characteristics (pain DETECT), catastrophizing thoughts scale (CTS), depression anxiety and stress scale (DASS), pain pressure threshold (PPT)	NRS (0-10)	Patients with chronic postoperative pain (NRS≥3) had a higher BMI, higher scores on the DASS and CTS compared with patients with a NRS <3
George 2022	Retrospective cohort	1146	6 months	Age, sex, race, BMI, tobacco use, comorbidities, preoperative pain ratings	Graded chronic pain scale	Variables associated with high-impact pain were non-white race, two or more comorbidities, age less than 65year, preoperative pain scores 5/10. Female sex, smoking and non-white race were associated with bothersome chronic pain
Hardy 2022	Prospective cohort	96	12 months	Main evaluating criterium: PCS. Secondary evaluation criteria: Beck depression inventory (BDI), Geriatric depression score (GDS), stait-trait inventory anxiety (STAI-A/B), SF12 QoL, WOMAC	VAS (0–100), WOMAC	The PCS score was significantly correlated with pain at one year. Preoperative pain >60/100 and a trait anxiety score >46 were considered risk factors after multivariate analysis.
Hofstede 2018	Pooled analysis prospective cohorts	1491	12 months	Age, sex, BMI	HOOS, WOMAC, OHS, VAS	Age, BMI and female sex were associated with more postoperative pain after THA.
Lu 2021	Retrospective cohort	612	6 months	Sex, age, BMI, education, smoking, alcohol abuse, preoperative pain, depression, anxiety	NRS (1-10)	Preoperative pain, depression state, surgical type, acute postoperative pain and analgesic type were independent risk factors
Omran 2024	Retrospective cohort	1249	2 years	Sex, race, age, tabacco use, BMI, ASA score	Patient-reported outcomes measurement information system (PROMIS) pain intensity questionnaires	Higher BMI was associated with maintaining high pain over 2 years
Paredes 2025	Prospective cohort	103	6 months	Sex, age, BMI, number of comorbidities, pain duration (months), brief pain inventory (BPI 0–10), WOMAC, HADS, life orientation test-revised (LOT-R), satisfaction with life scale (SWLS)	Cluster membership (1-2-3) based on pain intensity (BPI), pain interference (BPI) and disability (WOMAC)	Preoperative anxiety was a predictor for cluster 3 membership, functional disability for cluster 2 membership.
Singh 2010^a^	Prospective cohort	5707 3289	3 years 5 years	Age, sex, BMI, comorbidities (Deyo-Charlson score), depression and anxiety (ICD-9 code)	Mayo hip score: Moderate or severe pain	At 2 years patients with BMI >35 and depression had higher odds, and at 5 years all BMI categories >25 had higher odds of moderate-severe hip. Sex, age comorbidities and anxiety were not associated with moderate-severe pain.
Singh 2013	Prospective cohort	5707 3289	3 years 5 years	Heart disease, peripheral vascular disease, renal disease, COPD, Diabetes, Connective tissue disease	Mayo hip score: Moderate or severe pain	Peripheral vascular disease had a non-significant association with pain at 2 years while renal disease seemed protective
Tang 2023	Prospective observational study	80	3 months	Age, sex, BMI, education level, medical history, preoperative pain (NRS), PSQI	NRS (1-10)	No risk factors of chronic pain after THA were identified
Ueki 2025	Retrospective cohort	311	12 months	Preoperative pain intensity (NRS), neuropathic pain via the painDETECT questionnaire, central sensitization inventory (CSI), PCS	NRS (0–10), persistent pain group (NRS score ≥3)	Preoperative neuropathic pain, central sensitization and pain catastrophizing were associated with persistent pain in the univariate analysis. Logistic regression analysis showed an independent association of central pain sensitization and catastrophizing
Wylde 2024	Prospective/cross sectional cohort	254	12 months	Preoperative widespread pain (PPT on the pain free volar forearm)	WOMAC	Strong association between preoperative pressure pain threshold and pain severity 12 months after surgery.

THA, Total Hip Arthroplasty; TKA, Total Knee Arthroplasty, OA osteoarthritis, WOMAC, Western Ontario and McMaster Universities osteoarthritis index; ASA; American Society of Anesthesiologists (Physical Status classification), VAS, Visual Analogue Scale; NRS, Numeric Rating Scale; BMI, Body Mass Index; COPD, Chronic Obstructive Pulmonary Disease.

a Both these studies used the same study cohort whose data was collected at 2- and 5-years post-surgery. Demographics from both measuring moments were included.

### 3.2 Assessment of quality of the included studies

**Fig. 2:**
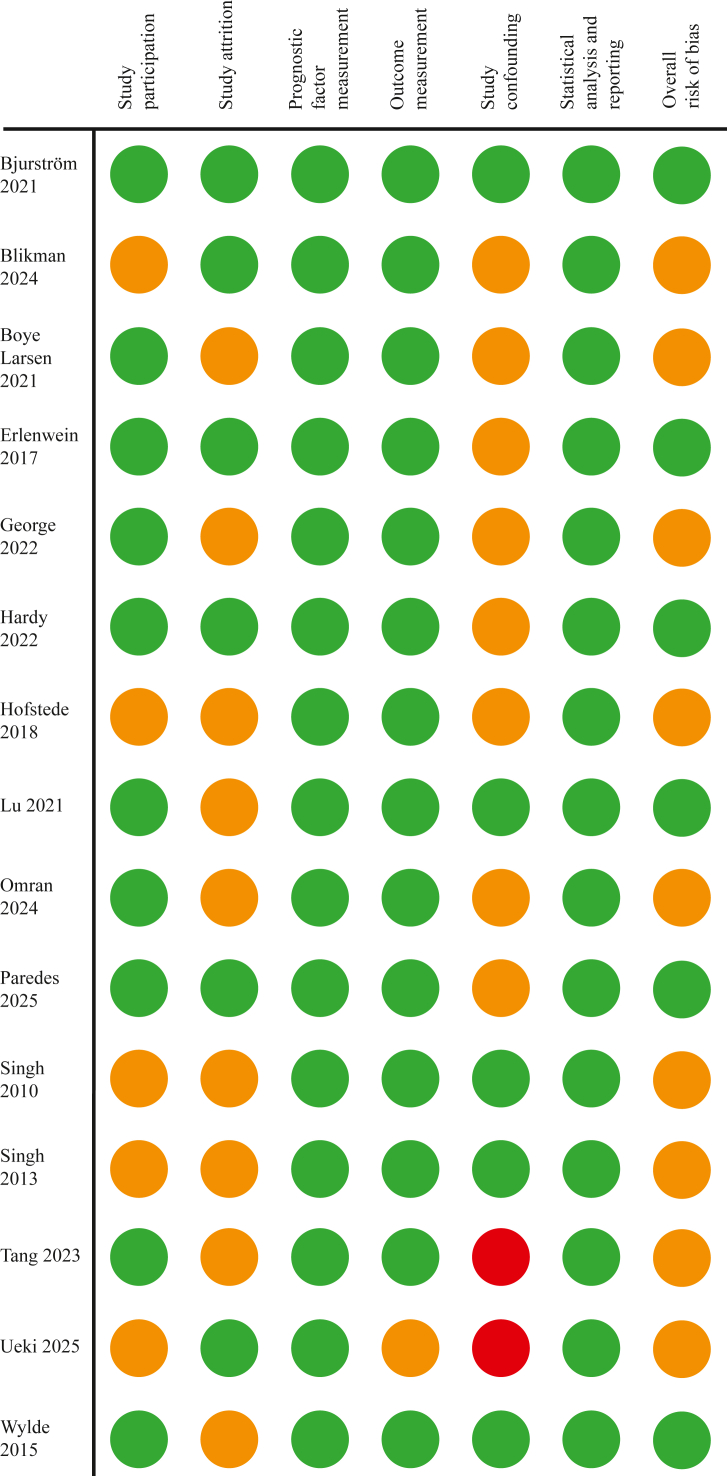
Bias assessment.

Six studies had low overall risk of bias. Nine studies had moderate risk of bias, mainly due to study attrition, confounding, and outcome measures. Two studies were rated moderate-to-high risk of bias for study confounding. (Fig. 2; Supplement C).

### 3.3 Preoperative risk factors in developing chronic pain after THA

**Fig. 3:**
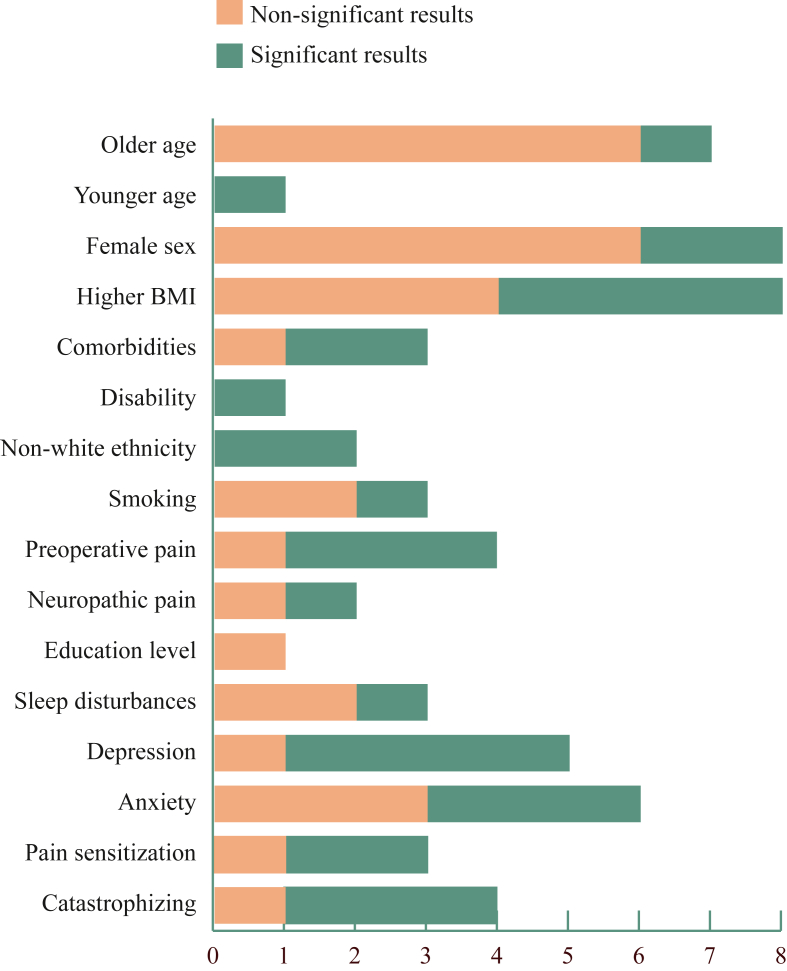
Number of studies reporting on the included domains.

Fig. 3 shows the domains studied and their significant findings. Detailed results are presented in Table 2. GRADE assessment was stated ‘low’ due to the observational character of all studies and was therefore not specified.

**Table 2:** Overview of significant results per domain.

Predictive factor	Studies showing significant results	Patients (n ​= ​)	Univariate or multivariable analysis	Results
**Biological**				
Older age	Hofstede 2018	1492	Multivariable	One-year increase in age resulted in more post-operative pain of 0.1 on scale of 0–100
Younger age	George 2022	1146	Multivariable	Age less than 65 years is associated with high impact chronic pain after surgery
Female sex	Hofstede 2018	1492	Multivariable	Being female was associated with more pain at 12 months post-surgery of 2 points on a scale of 0–100
	George 2022	1146	Multivariable	Women had an increased risk of developing bothersome chronic pain 6 months after surgery
Higher BMI	Erlenwein 2017	104	Univariate	Patients with chronic pain after 6 months had a significant higher BMI than the patients without chronic pain
	Hofstede 2018	1492	Multivariable	1-Point increase in in BMI resulted in more postoperative pain of 0.36 on a scale of 0–100
	Singh 2010	57073289^a^	Multivariable	At 2-years a BMI >35 and at 5 years a BMI >25 was associated with higher odds chronic pain
	Omran 2024	1249	Multivariable	Higher BMI is associated with maintaining high pain over 2 years after THA
Comorbidities	George 2022	1146	Multivariable	Having two or more comorbidities was associated with developing chronic pain after surgery
	Singh 2013	57073289	Multivariable	Peripheral vascular diseases increase the odds of pain 2-years after surgery, but not 5-years postoperatively. Renal disease was protective of developing chronic pain.
Disability	Paredes 2025	103	Multivariate	Preoperative functional disability was a predictor for cluster 3 membership, compared to cluster 1 (clusters ranging from 1 to 3, based on pain profiles)
Non-white race	George 2022	1146	Multivariable	Association with high impact and bothersome chronic pain
	Omran 2024	1249	Multivariable	African americans had slightly higher odds to maintain pain over a 2-year trajectory instead of recovering within a month
Smoking	George 2022	1146	Multivariable	Association with bothersome chronic pain
Preoperative pain	George 2022	1146	Multivariable	Pain scores of 5/10 or higher were associated with high impact chronic pain
	Hardy 2022	96	Multivariable	Preoperative pain was associated with the development of chronic pain after surgery
	Lu 2021	612	Multivariable	Preoperative pain in the surgical area was associated with the development of chronic pain.
	Blikman 2024	245	Univariate and multivariable	Preoperative neuropathic-like symptoms were associated with higher oxford hip scores and higher pain scores (NRS) 12 months after surgery
Pain sensitization	Wylde 2014	254	Multivariable	Higher pain sensitization was strongly associated with chronic pain after 12 months
	Ueki 2025	311	Univariate and multivariable	Central sensitization was associated with a higher pain intensity (NRS) and more persistent pain at 12 months postoperatively
**Psychological**				
Sleep disturbance	Bjurström 2021	52	Multivariable	Sleep disturbance is associated with pain 6 months after surgery
Depression	Erlenwein 2017	104	Univariate	Preoperative depression is associated with a higher risk of chronic pain after surgery
	Lu 2021	612	Multivariable	Preoperative depression is associated with a higher risk of chronic pain after surgery
	Singh 2010/2013	57073289	Multivariable	Presence of the ICD-9 code of depression increased the odds of chronic pain 2-years post-surgery
Anxiety	Erlenwein 2017	104	Univariate	Patients with chronic pain 6 months after surgery had higher anxiety scores compared to patients without pain
	Hardy 2022	96	Multivariable	A preoperative trait anxiety score >46 was a risk factor for pain >30/100 1 year after THA
	Paredes 2025	103	Multivariable	Preoperative anxiety was a predictor for cluster 3 membership, compared to cluster 1 (clusters ranging from 1 to 3, based on pain profiles)
Pain catastrophizing	Hardy 2022	96	Univariate	Significant results in the relationship between pain catastrophizing and the development of pain 1-year post-surgery
	Erlenwein 2017	104	Univariate	Patients with chronic pain 6 months after surgery had higher scores on the catastrophizing thoughts scale compared to patients without pain
	Ueki 2025	311	Univariate and multivariable	Pain catastrophizing was associated with a higher pain intensity (NRS) and more persistent pain at 12 months postoperatively

a Number of participants differed at 2- and 5-years post-surgery.

#### 3.3.1 Biological patient characteristics

Eight studies reported on the association between age and chronic pain after THA. One study demonstrated a rise in pain of 0.18 on a 0–100 scale for each additional year in age [17]. Another study identified age below 65 years as a risk factor for high impact CPSP [19]. Six studies did not find a significant association [6,20,[22], [23], [24], [25]].

One study reported that women experienced more pain 12 months post-surgery, with a mean increase of 2 points on a 0–100 scale [17]. Another study demonstrated that 6 months after surgery, women more often experience bothersome chronic pain, but not high impact chronic pain [19]. Six studies reported no significant differences between sexes [6,20,[22], [23], [24], [25]].

Eight studies included Body Mass Index (BMI). One study reported significantly higher BMI in patients with a Numeric Rating Scale (NRS) pain score >3 (mean BMI 30.8) at 6 months postoperatively compared to those with a NRS <3 (mean BMI 27.4) [22]. Another study found that for each 1-point increase in BMI, pain scores increased by 0.36 points on a scale of 0–100 ​at 12 months after surgery [17]. Singh et al. categorized BMI into multiple groups and assessed pain outcomes at two postoperative follow up moments. At 2-years, a BMI >35 was significantly associated with increased odds of moderate to severe pain. At 5 years, all BMI categories >25 were linked to moderate to severe pain, with higher BMI categories showing progressively elevated odds [23]. A fourth study demonstrated that each unit increase in BMI was associated with a 12 ​% higher risk of CPSP at 2 years [24]. Two studies reported no significant association between BMI >30 and chronic pain outcomes [6,19]. One study did not find differences in BMI between pain clusters and one study did not find BMI as an independent risk factor in multivariate regression analysis [20,26].

One study linked tobacco use to bothersome pain at six months but not to high-impact pain [19]. Two studies found no significant association [6,24].

Patients who do not identify as White were more likely to develop high impact and bothersome CPSP at 6 months compared to White patients, in one study [19]. Another study demonstrated higher odds in African Americans (OR 3.18) for CPSP after 2 years [24].

Two studies found a significant influence of preoperative pain scores of >5/10 and ​> ​60/100 on the development of high impact chronic pain [19,27]. Another study also found an association of preoperative surgical-site pain with CPSP after THA, while an association of other preoperative pain was not fount [6]. One study did not find a significant correlation [22].

One study used as score of ≥13 on the modified painDETECT questionnaire (mPDQ) to discriminate between nociceptive pain and neuropathic-like symptoms. Preoperative neuropathic-like symptoms was linked to moderate and severe pain on the Oxford Hip Score and pain at rest and with movement (NRS ≥1) in a multivariate logistical model [25]. One study defined a score of ≥13 on the painDETECT as an indicator for neuropathic pain symptoms, and found an association between preoperative neuropathic pain and persistent pain (NRS ≥3) in a univariate analysis, but not after logistic regression analysis [26].

Preoperative pain sensitization was studied in three studies. Widespread pain sensitivity was measured by assessment of forearm pain pressure threshold (PPT) at the pain free volar forearm. Lower PPT indicates a higher pain sensitivity, and was strongly associated with CPSP after 12 months in one study [18]. Another study found differences in preoperative PPT in patients with CPSP after 6 months compared to patients without [22]. The third study used the Central Sensitization Inventory (CSI), for identifying central sensitization symptoms. A score of 40 or more was an independent risk factor for CPSP 12 months after THA, with a NRS >3 [26].

One study assessed disability with a subscale of the Western Ontario and McMaster Osteoarthritis Index (WOMAC), with a higher score indicating more disability. Patients were allocated in three clusters, based on pain intensity, interference and disability. They found a significant association between disability and cluster 2, with every unit increase on the WOMAC, there was 4 ​% higher odds of being in cluster 2 compared to cluster 1. This association was not found for cluster 3 [20].

Three studies mentioned comorbidity. One study used the Deyo-Charlson score which consists of a weighted scale of 17 comorbidities and uses the outcome of this score as a continuous variable for their analyses [23]. The combination of comorbidities did not demonstrate a significant effect on CPSP. When comorbidities are examined separately, it was found that peripheral vascular disease had a significant effect on the development of pain 2-years after surgery, but not after 5-years. Renal disease was associated with less moderate to severe pain after 2 years. No association was found for the other comorbidities examined [28]. Another study demonstrated that having two or more comorbidities was associated with CPSP, but individual comorbidities were not specified [19].

#### 3.3.2 Socioeconomic patient characteristics

One study briefly mentions education. When using the baseline characteristics, a univariate analysis found no significant effect of the education level on CPSP at 6 months [6].

#### 3.3.3 Psychological patient characteristics

Three studies assessed preoperative sleep quality. One study found that poor sleep quality, with a score of >5 on the Pittsburgh Sleep Quality Index (PSQI, 0–21), was significantly associated with CPSP at 6 months postoperatively. Higher PSQI scores correlated with increased pain intensity 6 months postoperatively, on both the brief pain inventory short form as the WOMAC pain score [29]. Two studies found no significant association between preoperative sleep quality and CPSP [21,30].

Three studies linked depressive symptoms to CPSP at 6 months [6,20,22]. A higher preoperative score on the Center for Epidemiological Studies-Depression scale was a predictor in one study [6]. Another study used the Depression Anxiety and Stress Scale (DASS) to demonstrate a similar association [22]. The third study correlated higher preoperative scores on the Hospital Anxiety and Depression Scale (HADS) to more severe chronic pain clusters at six months [20]. Another study demonstrated that the presence of an International Classification of Diseased-ninth revision (ICD-9) code of depression preoperatively increased the odds of developing pain 2-years post-surgery with 2.1. At 5-years post-surgery this association was not found in a smaller population (3289/5707) [23]. One study found no association between preoperative depression (HADS) and CPSP [21].

Six studies evaluated anxiety. Preoperative anxiety (DASS) was associated with CPSPS at 6 months in one study [22]. A trait anxiety score >46 was a risk factor for pain with an intensity of >30/100 after 1 year in another study [27].Another study found that preoperative anxiety (HADS), was linked to the highest pain cluster (cluster 3), but not to the cluster 2 pain profile. Each unit increase, increased the likelihood of belonging to cluster 3 by 18 ​% and 13 ​% compared to cluster 1 or 2 [20]. Three studies found no associations between preoperative anxiety and CPSP after THA [6,21,23].

Preoperative pain catastrophizing was assessed using the Pain Catastrophizing Scale (PCS) in three studies. One study found that a PCS ≥19/52 is a significant predictor for CPSP at 12 months [27]. Another study reported that PCS >30 is an independent risk factor for CPSP at 12 months [26]. The third study found no significant association [21]. One study found a higher preoperative score on the Catastrophizing Thoughts Scale (CTS) in patients with chronic pain compared to patients without chronic pain, 6 months after THA [22]. Another study found an association between a higher score on the Coping Strategies Questionnaire-Revised with a more severe pain cluster at 6 months after surgery, although this relationship did not persist after logistic regression analysis [20].

## 4 Discussion

This review offers insights for perioperative optimization and guided research by integrating the biopsychosocial domains on risk factors for CPSP after THA.

The multiple associated factors demonstrate the complexity of the CPSP pathophysiology. These predictive factors include (younger) age, female sex, higher BMI, having multiple comorbidities, preoperative (neuropathic) pain, smoking, non-white race, sleep disturbance, depression, anxiety, pain sensitization and pain catastrophizing. Previous data published before the cutoff point of our inclusion criteria supports these findings [8,9].

There are different approaches for performing a THA, with their own advantages and disadvantages. Nerve injury occurs in around 1 ​% of the patients. The anterior approach has a higher occurrence rate of neuropraxia of the lateral femoral cutaneous nerve, but rarely leads to chronic neuropathic pain [31]. Novel minimal invasive techniques did not reduce CPSP after THA. Revision surgery was not associated with more CPSP in the study of George et al. [32] Surgical approach does not seem to have an effect on postoperative opioid use as well [33]. Studies on acute postoperative pain after THA found ambivalent results regarding CPSP [6,34,35].

This review includes literature since 2010, reflecting the advances in surgical techniques and standardized multimodal anaesthesia protocols for THA [36,37]. Since then, the incidence of CSPS did not significantly decrease, indicating that other risk factors are associated with CPSP [3,38]. Altering these risk factors might improve outcome after THA. This review highlights the complexity of chronic (postsurgical) pain, by addressing different domains of the biopsychosocial model.

### 4.1 Overall findings

#### 4.1.1 Biological patient characteristics

Although chronic pain is more prevalent in older adults, this was not substantiated in this review. Two out of eight studies identified age as a significant risk factor, with minimal, potentially clinically irrelevant, observed differences in pain scores. The high mean age and narrow age range in these osteoarthritis-dominated THA cohorts might attribute to this discrepancy [39]. One study found an association between younger age and chronic pain, which is supported by studies that suggest a higher risk of CPSP in younger patients [7,40].

Female sex is an established risk factor for chronic pain, with evidence indicating that women are more likely to experience and report chronic pain than men, with higher pain intensity, lower pain thresholds, and reduced pain tolerance [7,41]. Studies on other TJAs, particularly TKA, identified female sex as a risk factor for CPSP [19,42,43]. While two studies found an association, six studies found no difference between sexes. However, one of these studies reported an increased use of non-steroidal anti-inflammatory drugs and opioids among women at 2 and 5 years after THA [23]. Background variables were not analyzed by sex in the studies, limiting interpretation if risk factors are similar for men and women, as previous research on pain sensitivity suggests [44]. Gender identity can influence the experience and development of pain as well, but no studies included gender identity as a demographic factor.

Higher BMI was associated with developing CPSP in four of eight studies, with one study demonstrating increasing odds with increasing BMI [23]. Obesity (BMI >30) is known to increase chronic pain, possibly by placing strain on weight-bearing joints or reducing physical activity [7,45].

One study indicated a higher risk of CPSP after THA among patients not identifying as white, but the specific ethnicities were not detailed. Broader chronic pain research finds higher pain prevalences among Asian, Black or mixed-ethnic groups, compared to White people, but these differences diminish after adjusting for socio-economic factors [19,46]. There is some evidence supporting race as an independent risk factor for CPSP after THA, and some findings suggest that targeting other risk factors, might be more beneficial in the African American patients [24]. However, racial analyses are inherently complex, and most studies lack adjustment for confounders [47].

Multiple physical and mental chronic comorbidities are associated with chronic pain, assumingly through increased nociception from the periphery and dysregulation of the pain-modulatory circuitry [7,48]. This review supports this theory.

The relationship between preoperative pain and persistent pain has been described after other surgeries [49]. Neuropathic pain-like symptoms are present in approximately 29 ​% patients with hip osteoarthritis [50,51]. Two studies identified neuropathic-like pain as a prognostic factor for CPSP after THA [25,26]. This association was not observed after adjusting for central sensitization and pain catastrophizing in one study, highlighting the complexity and multifactorial nature of CPSP [26].

Higher pain sensitization appears to be an important prognostic factor, and is consistently associated with CPSP in general [44,52,53]. It remains unclear whether the severity of pain is influenced by pain sensitivity, or if the presence of chronic pain leads to higher pain sensitivity. Normalization of pain-processing tests after TJA have been demonstrated, suggesting that a higher pain sensitivity may be maintained by the presence of pain [54,55]. One study demonstrated an association of pain sensitization with CPSP 12 months after THA, but not after TKA, indicating different mechanisms in the development of CPSP [18].

#### 4.1.2 Socioeconomic patient characteristics

Socio-economic disadvantages are associated with an increased risk of chronic pain. People with lower-level education or lack of work autonomy experience higher levels of chronic pain [56,57]. Possible mechanisms are less effective coping strategies or lack of a social support system [58]. It can be assumed that socio-economic risk factors might influence CPSP after THA, but only one study reported on education.

#### 4.1.3 Psychological patient characteristics

One study found a significant relationship between sleep disturbances and CPSP, and opioid use after surgery [29]. Literature on CPSP supports this, where preoperative sleep deprivation is found to be a significant factor [59]. Sleep deterioration is also associated with chronic pain in general [60]. Another large observational study shows that (the level of) postoperative sleep disturbances are also strongly associated with (the severity of) chronic pain [61]. Additional studies must be conducted to verify if perioperative sleep interventions may enhance postoperative outcome after THA.

Depression is a significant risk factor for CPSP after THA in four out of five studies. Depression leads to higher pain severity and more pain sites [62]. This also applies to other interventions [63]. Anxiety is an established predictor for chronic (postoperative) pain as well [62,63]. Contradictory results are presented in this review, possibly because of the lower prevalence of anxiety disorders in higher age [64]. Self-report measures on anxiety in elderly can also be incongruent because of differences in experiences or interpretation of affective terms [65].

The association of pain catastrophizing in this review, is supported by literature. Catastrophizing is associated with higher pain levels, increased pain interference and is a predictive factor for CPSP in general [63,66].

Post-traumatic stress disorder is a risk factor for worse functional outcome and quality of life, especially at a younger age, after TJA [67]. The influence on CPSP is not yet investigated to our knowledge [68].

### 4.2 Implications for clinical practice and future research

Targeted preoperative optimalisation may reduce CPSP after THA. Quantitative sensory testing could identify high-risk patients [42]. Further research should focus on patient selection and the effect of the used interventions [69]. Broad prehabilitation might benefit all patients, as better preoperative health-related quality of life correlates with better postoperative outcome [17]. Weight loss and smoking cessation should be encouraged, potentially reducing both chronic pain as other perioperative complications [[70], [71], [72]]. Perioperative psychological interventions improving mental health and optimizing sleep hygiene show promising results on CPSP [73]. Digital applications could improve adherence to these interventions [74,75].

Perioperative optimalisation of analgesia could also be a target in high risk patients, as multimodal anesthetic strategies are effective in reducing acute pain after THA [76]. Its impact on CPSP remains unclear [77,78]. Femoral nerve blocks lack strong evidence on reducing CPSP, yet adding them to multimodal, opioid sparing regimes in high-risk patients may improve outcome [36,79]. Emerging locoregional techniques, such as the Quadratus lumborum of Pericapsular Nerve Group block, may offer superior alternatives [80,81]. Adding gabapentinoids, such as pregabalin, or serotonin norepinephrine reuptake inhibitors, such as duloxetine, show mixed results overall but may benefit patients with central sensitization [77,82].

Recognition of non-modifiable factors in the preoperative process is vital and should be included in the informed consent. Denervation of the hip joint might serve as an alternative for pain relief, improving function and quality of life in patients with chronic hip pain [83,84].

Motivating patients for prehabilitation or conservative treatments can be challenging. Most patients with osteoarthritis experience their hip pain as ‘pathoanatomically’ with the preposition that surgery is inevitable. They fear that activity might damage their hip, accelerating the process. Providing detailed education can improve the effectuation of conservative treatment strategies [85]. Tailored strategies, for example providing reminders, allowing more flexibility, and adding representatives in the treatment team, may improve adherence in underrepresented populations [86]. Suboptimal rehabilitation trajectories also impair postoperative outcome, highlighting the importance of motivating patients and securing access to rehabilitation facilities throughout the whole perioperative trajectory [24].

Although not included in our review, reducing preoperative opioid use could be a target as well. Opioid use in the 90 days before surgery increases the risk of chronic opioid use after THA [33,87]. In studies on other TJAs, preoperative opioid use was the strongest predictor for persistent opioid use. Other identified risk factors were a younger age, associated backpain, chronic pain syndromes and comorbidities, such as Ehlers Danlos syndrome and COPD [[88], [89], [90]]. Education on expectation management show promising results on the reduction of postoperative opioid consumption [91,92].

### 4.3 Strengths and limitations

Several limitations must be acknowledged. Many studies did not adjust for confounders, and attrition bias was common due to low follow-up rates and missing data on dropouts. Data on some factors was limited, resulting in low evidence. Heterogeneity in study design, pain definitions, and assessment tools precluded meta-analysis and may explain the inconsistent findings. All included studies employed observational cohort designs, resulting in low overall quality.

Publication bias may exist, as studies reporting significant associations are more likely to be published.

Excluding grey literature potentially omitted relevant data, though the selected databases cover most published research. At least two reviewers conducted each step, applying standardized risk-of-bias thresholds to minimize assessment bias.

## 5 Conclusion

In conclusion, CPSP after THA is a multifactorial condition that warrants a comprehensive biopsychosocial approach. Key predictive factors include BMI, preoperative (neuropathic) pain, central sensitization, multiple comorbidities, smoking, non-white race, sleep disturbances, depression, anxiety, and pain catastrophizing. Age, especially younger, and female sex might also contribute to CPSP after THA. Although the observational nature limits the evidence, several studies included a high number of patients and findings are consistent with literature on CPSP and chronic pain. The combination of risk factors can result in clinically relevant increase of the overall risk, even when individual effect sizes are modest. CPSP influences the quality of life and functional outcome in patients, with limited treatment options. Low-impact preventive strategies could reduce the prevalence and intensity of CPSP, including targeted preoperative prehabilitation and personalized perioperative management. In high-risk patients, conservative treatment should be encouraged and alternative pain interventions might be considered. Graphical abstract summarizes the results and future perspectives.

## Author contributions

All authors made substantial contributions to the work and approved this version.

Rachel Smits is the first and corresponding author and responsible for conceptualization, the methodology, formal analysis, visualization, project administration and writing the article.

Rosa Poppen was responsible for the investigation, resources and data duration, formal analysis and preparation of the original draft.

Jetze Visser was responsible for the validation and reviewing and editing of the writing.

Kris Vissers was responsible for the validation and review and editing of the writing.

Selina van der Wal was responsible for the supervision, validation, conceptualization, reviewing and editing of the writing.

## Data statement

The used data is added in the supplemental document.

## Funding

The authors have no sources of funding to declare for this manuscript.

## Conflict of interest

The authors do not have any conflicts of interest to declare.

## GLOSSARY OF TERMS

CPSP: Chronic postsurgical pain
THA: Total Hip Arthroplasty
QUIPS: Quality in Prognostic Studies
TJA: Total Joint Arthroplasties
TKA: Total Knee Arthroplasty
TSA: Total Shoulder Arthroplasty
BMI: Body Mass Index
NRS: Numeric Rating Scale
mDPQ: modified painDETECT questionnaire
OHS: Oxford Hip SCore
PPT: Pain Pressure Threshold
CSI: Central Sensitization Inventory
HRQol: Health Related Quality of life
WOMAC: Western Ontario and McMaster Universities osteoarthritis index
PSQI: Pittsburgh Sleep Quality Index
BPI-sf: Brief pain inventory short form
DASS: Depression Anxiety and Stress Scale
HADS: Hospital Anxiety and Depression Scale
PCS: Pain Catastrophizing Scale
CTS: Catastrophizing Thoughts Scale
CSQ-R: Coping Strategies Questionnaire-Revised
