# Supplementary material for: Biopsychosocial predictive factors for developing chronic postsurgical pain after hip replacement surgery: A systematic review
Source: Osteoarthr Cartil Open. 2025 Dec 4;8(1):100725. doi: 10.1016/j.ocarto.2025.100725 (PMC12765098; doi:10.1016/j.ocarto.2025.100725)
Supplement: Multimedia component 1 [file mmc1.docx]

## **Supplement A Search strategies**

**PubMed Search**

"Chronic Pain"[MeSH Terms] OR

"pain threshold"[MeSH Terms] OR

"neuralgia"[MeSH Terms] OR

"pain, intractable"[MeSH Terms] OR

chronic pain*[Title/Abstract] OR

intractable pain*[Title/Abstract] OR

nerve pain*[Title/Abstract] OR

neuralgia*[Title/Abstract] OR

neurodynia*[Title/Abstract] OR

neuropathic pain*[Title/Abstract] OR

pain threshold*[Title/Abstract] OR

refractory pain*[Title/Abstract] OR

(chronic*[Title/Abstract] AND widespread pain*[Title/Abstract])

OR

(("Chronic Pain"[MeSH Terms] OR

"Chronic Disease"[MeSH Terms] OR

"Time Factors"[MeSH Terms] OR

chronic*[Title/Abstract] OR

Widespread[Title/Abstract] OR

CWP[Title/Abstract] OR

neuralgia*[Title/Abstract] OR

nerve pain*[Title/Abstract] OR

Intractable[Title/Abstract] OR

Refractory[Title/Abstract] OR

threshold*[Title/Abstract] OR

time factor*[Title/Abstract] OR

persistent[tiab] OR

preoperative determinant*[tiab])

AND

("pain, postoperative"[MeSH Terms] OR

postoperative pain*[Title/Abstract] OR

post operative pain*[Title/Abstract] OR

post-surgical pain*[Title/Abstract] OR

postsurgical pain*[Title/Abstract]))

AND

("arthroplasty, replacement, hip"[MeSH Terms] OR hip arthroplast*[Title/Abstract] OR

hip prosthesis implant*[Title/Abstract] OR

hip surger*[Title/Abstract] OR

hip replacement*[Title/Abstract])

**EMBASE search**

exp hip arthroplasty/ OR (Hip arthroplast* OR Hip Prosthesis Implant* OR Hip surger* OR Hip Replacement*).ti,ab,kf.

AND

(Chronic Pain/ OR (Chronic Pain* OR Chronic* Widespread Pain*).ti,ab,kf. OR exp Neuralgia/ OR (Neuralgia* OR Neuropathic Pain* OR Neurodynia* OR Nerve Pain*).ti,ab,kf. OR intractable pain/ OR (Intractable Pain* OR Refractory Pain*).ti,ab,kf. OR Pain threshold/ OR (pain threshold* OR pain tolerance).ti,ab,kf. OR

Chronic Pain/ OR exp Chronic Disease/ OR Time Factor/ OR (Chronic* OR widespread OR CWP OR neuralgia* OR nerve pain* OR intractable OR refractory OR threshold* OR time factor* OR persistent OR preoperative determinant*).ti,ab,kf.

AND

Postoperative pain/ OR (postoperative pain* OR post-operative pain* OR post -surgical pain* OR postsurgical pain*).ti,ab,kf.

Limit to conference abstract status AND Limit to (dutch or english)

**CENTRAL Search**

ID Search

#1 (chronic pain):ti,ab,kw

#2 MeSH descriptor: [Arthralgia] explode all trees

#3 MeSH descriptor: [Neuralgia] explode all trees

#4 MeSH descriptor: [Chronic Pain] explode all trees

#5 neuropathic pain

#6 nerve pain

#7 neuralgia

#8 refractory pain

#9 persistent

#10 #1 OR #2 OR #3 OR #4 OR #5 OR #6 OR #7 OR #8 OR #9

#11 MeSH descriptor: [Arthroplasty, Replacement, Hip] explode all trees

#12 hip replacement

#13 (hip arthroplasty):ti,ab,kw

#14 ("hip replacement arthroplasty"):ti,ab,kw

#15 hip prosthesis

#16 #11 OR #12 OR #13 OR #15

#17 MeSH descriptor: [Pain, Postoperative] explode all trees

#18 postoperative pain

#19 postsurgical pain

#20 post operative pain

#21 post-surgical pain

#22 #17 OR #18 OR #19 OR #20 OR #21

#23 #10 AND #16 AND #22
