# Supplementary material for: Biopsychosocial predictive factors for developing chronic postsurgical pain after hip replacement surgery: A systematic review
Source: Osteoarthr Cartil Open. 2025 Dec 4;8(1):100725. doi: 10.1016/j.ocarto.2025.100725 (PMC12765098; doi:10.1016/j.ocarto.2025.100725)
Supplement: Multimedia component 2 [file mmc2.docx]

**Supplement B. Overview of study characteristics and results**

| **Article and year** | **Country** | **Design** | **Patients (n=)** | **Age** | **Female**  **(%)** | **Follow up** | **Inclusion** | **Exclusion** | **Factors included** | **Outcome measurement** | **Confounders** | **Statistical analysis** | **Results** |
| --- | --- | --- | --- | --- | --- | --- | --- | --- | --- | --- | --- | --- | --- |
| ***Bjurström 2021*** | Sweden | prospective cohort | 52 | 70.4 | 60 | 6 months | age ≥18 years, OA pain du- ration ≥12 months, average pain intensity ≥4/10 , THA conducted under spinal anesthesia | acute illness, malignant disease, ASA>3, any contraindications to lumbar puncture, immunomodulating treatment | Pittsburgh sleep quality index (PSQI), brief pain inventory short form (BPI-sf), douleur neuropathique 4 (DN4), pain catastrophizing scale (PCS), Western Ontario and McMaster Universities osteoarthritis index (WOMAC OA), hospital anxiety and depression scale (HADS) | BPI-SF (0-10), WOMAC | demographic factors (age, BMI), preoperative PCS score and HADS score | Linear regression models | preoperative sleep disturbance was associated with higher pain severity 6 months after THA |
| ***Blikman 2024*** | The Netherlands | Prospective cohort | 245 | 70.4 | 69.8 | 12 months | Primary THA | Secondary OA, cognitive impairment | Age, sex, BMI, preoperative pain intensity (NRS 0-10) and duration (months), Neuropathic-like symptoms via the modified painDETECT Questionnaire (mPDQ) | Oxford Hip Score (OHS), pain (NRS 0-10) |  | Univariate and multivariate logistic regression | Preoperative neuropathic-like symptoms were a predictor for an unfavorable long-term pain outcome |
| ***Boye Larsen 2021*** | Denmark | retrospective cohort, secondary analysis from a larger randomized controlled trial | 89 | x | x | 12 months | Patients included in an randomized controlled trial on postoperative administration of chlorzoxazone (muscle relaxant; no effect). | use of gabapentinoids, glucocorticoids, opioids, anxiolytics, antiepileptics or antidepressants; alcohol abuse; other pain treatments outside of standard care; malignant conditions; pregnancy; BMI > 40 kg/m2; peripheral or central acting diseases; allergy towards chlorzoxazone; perioperative complications (e.g. fractures) and liver diseases. | sleep quality (PSQI), pain catastrophizing scale (PCS), hospital anxiety and depression scale (HADS), pain in hip (VAS 0-10) | Pain (VAS 1-10) |  | linear regression model | Preoperative PCS, anxiety, depression and PSQI were no independent risk factors for chronic pain postoperatively |
| ***Erlenwein 2017*** | Germany | Prospective cohort | 104 | 63 | 58 | 6 months | 18 years old and have sufficient knowledge of the German language. Only patients undergoing primary endoprosthetic surgery of the hip were included | a neuraxial or regional anesthesia, history of drug abuse, and acute hip pain caused by necrosis of the femoral head, the occurrence of a postoperative delirium syndrome, or surgical complications. | Age, Sex, BMI, pain history (German Pain Questionnaire), preoperative medication use (MQS), severity of chronic pain (CPG), chronicity of patients condition (MPSS), preoperative hip pain (NRS 0-11), neuropathic characteristics (pain DETECT), Catastrophizing Thoughts scale (CTS), Depression Anxiety and Stress Scale (DASS), Pain pressure threshold (PPT) | Pain (NRS 0-11) |  | univariate | Patients with chronic postoperative pain (NRS≥3) had a higher BMI, higher scores on the DASS and CTS compared with patients with a NRS < 3 |
| ***George 2022*** | USA | Retrospective cohort | 1146 | x | 54 | 6 months | Patients who underwent THA identified by Current Procedural Terminology (CPT) code | Died prior to end study, opted out of being contacted, did not have email | Age, sex, race, BMI, tobacco use, comorbidities, preoperative pain ratings | Graded Chronic Pain Scale |  | logistic regression analysis | Variables associated with increased risk on high-impact pain were non-white race, two or more comorbidities, age less than 65year, preoperative pain scores 5/10. Female sex, smoking and non-white race were associated with bothersome chronic pain |
| ***Hardy 2022*** | France | Prospective cohort | 96 | 69.7 | 65 | 12 months | all patients undergoing THA, >18 years of age, autonomous and living at home | previous surgery, absence of consent, patient unable to understand questionnaires | Main evaluating criterium: pain catastrophizing scale (PCS) Secondary evaluation criteria: Beck Depression Inventory (BDI), Geriatric Depression score (GDS), Stait-Trait Inventory anxiety (STAI-A/B), SF12 QoL, WOMAC | Pain (0-100), Western Ontario and McMaster Universities osteoarthritis index (WOMAC) |  | logistic regression analysis | The PCS score was significantly correlated with pain at one year, Preoperative pain > 60/100 and a trait anxiety score > 46 were considered risk factors after multivariate analysis. |
| ***Hofstede 2018*** | The Netherlands | Pooled analysis prospective cohorts | 1491 | x | x | 12 months | patients with primary THA with at least one preoperative and one postoperative measurement, with follow up > 12 months | cohorts regarding metal-on-metal prostheses | Age, Sex, BMI | Hip disability and osteoarthritis Outcome Score (HOOS), Western Ontario and McMaster Universities osteoarthritis index (WOMAC), Oxford Hip Score (OHS), Pain (VAS) |  | linear mixed models on each cohort separately | Age, BMI and female sex were associated with more postoperative pain after THA. |
| ***Lu 2021*** | China | retrospective cohort | 612 | 75.7 | 56 | 6 months | aged >65 years, clear indication of THA, combined spinal and epidural anesthesia | severe cardiovascular, chronic respiratory or central nervous system diseases, severe allergies, drug addiction, the use of antidepressants or sedative drugs, severe postoperative complications | Sex, Age, BMI, Education, Smoking, Alcohol abuse, preoperative pain, depression, anxiety | Pain (NRS 1-10) |  | multiple logistic regression analysis | preoperative pain, depression state, surgical type, acute postoperative pain and analgesic type were independent risk factors for chronic pain |
| ***Omran 2024*** | USA | Retrospective cohort | 1249 | 66 | 62 | 2 years | All patients who underwent THA and completed the Patient-reported outcomes measurement information system (PROMIS) pain intensity questionnaires | Experienced revison surgery, bilateral surgery, surgery involving multiple joints, trauma surgery | Sex, Race, Age, Tabacco use, BMI, ASA score | PROMIS |  | Growth mixture models | Higher BMI is associated with maintaining high pain over 2 years |
| ***Paredes 2025*** | Portugal | Prospective observational cohort | 103 | 66.6 | 54.5 | 6 months | Age > 50, unilateral total joint arthroplasty (TJA) due to osteoarthritis | Revision surgery, svere or disabling neurologic, psychiatric or organic disease, contralateral TJA in the previous six months | Sex, Age, BMI, number of comorbidities, pain duration (months), Brief pain inventory (BPI 0-10), Western Ontario and McMaster Universities osteoarthritis index (WOMAC), Hospital Anxiety and Depression scale (HADS), Pain Catastrophizing (CSQ-R) Life Orientation Test-Revised (LOT-R), Satisfaction with Life Scale (SWLS) | Cluster membership based on pain intensity (BPI), pain interference (BPI) and disability (WOMAC) | Surgery site | Post-hoc cluster analysis, multinominal logistic regression analysis | Preoperative anxiety was a predictor for cluster 3 membership, disability for cluster 2 membership. BPI, CSQ-R, HADS (depression) were associated with higher pain clusters in a post hoc test, this association was not found after multinominal logistic regression analysis |
| ***Singh 2010***** | United States of America | prospective cohort | 5707  3289 | 65  64.7 | 51  53 | 3 years  5 years | all patients who underwent THA and were alive at 5 year follow up |  | Age, Sex, BMI, comorbidities (Deyo-Charlson score), depression and anxiety (ICD-9 code) | Mayo Hip Score: moderate or severe pain | operative diagnosis, distance to medical center, implant type, ASA classification | univariate and multivariable-adjusted models (only ones presented) | At 2 years patients with BMI >35 and depression had higher odds, and at 5 years all BMI categories >25 had higher odds of moderate-severe hip. Sex, age comorbidities and anxiety were not associated with moderate-severe pain. |
| ***Singh 2013*** | United States of America | Prospective cohort | 5707  3289 | 65  64.7 | 51  53 | 3 years  5 years | all patients who underwent THA and were alive at 5 year follow up |  | Heart disease, peripheral vascular disease, renal disease, COPD, Diabetes, Connective tissue disease | Mayo Hip Score: moderate or severe pain | age, Sex, BMI, underlying diagnosis, ASA score, depression, anxiety, implant fixation, distance to medical center | univariate and multivariable-adjusted models | peripheral vascular disease had a non-significant association with pain at 2 years while renal disease seemed protective |
| ***Tang 2023*** | China | Prospective observational study | 80 | 72 | 62 | 3 months | Adults >65 years who had THA or TKA under general anesthesia | Trauma, patients with cognitive impairment or mental illness, severe hearing or language impairment, on orally administered central nervous system acting drugs | Age, Sex, BMI, Education level, Medical history, Preoperative pain (NRS), Preoperative sleep quality (PSQI) | Pain (NRS 0-10) |  | Chi-square test, Fisher’s exact tests, binary logistic regression models | No risk factors of chronic pain after THA were identified |
| ***Ueki 2025*** | Japan | Retrospective cohort | 311 | 66.1 | 78.1 | 12 months | THA for OA | Previous hip surgery, end-stage OA other joints, previous arthroplasty of the lower limb, spinal or neurological disease, use of pregabalin, gabapentin and duloxetine for pain | Preoperative pain intensity (NRS), neuropathic pain via the pain-DETECT (PD) questionnaire, Central Sensitization Inventory (CSI), Pain Catastrophizing Scale (PCS) | Pain (NRS 0-10), persistent pain group (NRS score ≥ 3) | Variables with a significance level of p<0.2 in the univariate analysis (BMI) | Univariate analysis (Mann-Whitney U and Chi-square), multivariate analysis (logistic regression analysis | Preoperative neuropathic pain, central sensitization and pain catastrophizing were associated with persistent pain in the univariate analysis. Logistic regression analysis showed an independent association of central pain sensitization and catastrophizing |
| ***Wylde 2024*** | United Kingdom | prospective/cross sectional cohort | 254 | 66.6 | 59 | 12 months | primary unilateral THA | inability to provide informed consent or complete questionnaires, medical comorbidities which precluded the use of spinal or locoregional anesthesia or strong opioid postoperatively | Preoperative widespread pain (Pressure pain threshold test pain free volar forearm) | Western Ontario and McMaster Universities osteoarthritis index (WOMAC) | age, sex, cohabitation, employment status, educational attainment, height and weight | linear regression models | strong association between pre operative pressure pain threshold and pain severity 12 months after surgery. |

THA, Total Hip Arthroplasty; TKA, Total Knee Arthroplasty, OA osteoarthritis, ASA; American Society of Anesthesiologists (Physical Status classification), VAS, Visual Analogue Scale; NRS, Numeric Rating Scale; BMI, Body Mass Index; COPD, Chronic Obstructive Pulmonary Disease

** Both these studies used the same study cohort whose data was collected at 2- and 5-years post-surgery. Demographics from both measuring moments were included.
