# Supplementary material for: Biopsychosocial predictive factors for developing chronic postsurgical pain after hip replacement surgery: A systematic review
Source: Osteoarthr Cartil Open. 2025 Dec 4;8(1):100725. doi: 10.1016/j.ocarto.2025.100725 (PMC12765098; doi:10.1016/j.ocarto.2025.100725)
Supplement: Multimedia component 3 [file mmc3.docx]

**Supplement B Bias assessment**

|  | Study participation | Study attrition | Prognostic factor measurement | Outcome measurement | | Study confounding | Statistical analysis and reporting |
| --- | --- | --- | --- | --- | --- | --- | --- |
| Bjurström 2021 | Low risk of bias | Low risk of bias | Low risk of bias | | Low risk of bias | Low risk of bias | Low risk of bias |
| Blikman 2024 | Moderate risk: non-responders were more often female, this could possibly mean gender-related bias, especially since female sex is associated with higher chronic pain risks | Low risk | Low risk | | Low risk | Moderate risk: the study adjusted for important known confounders (age, sex) However some potentially relevant confounders like psychological factors or comorbidities were not included | Low risk |
| Boye Larsen 2021 | Low risk of bias | Moderate risk of bias: included 48% of the original study population | Low risk of bias | | Low risk of bias | Moderate risk of bias: confounders are not specifically mentioned, but collinearity and VIF are. | Low risk of bias |
| Erlenwein 2017 | Low risk of bias | Low risk of bias | Low risk of bias | | Low risk of bias | Moderate risk of bias: confounders are not mentioned or processed in the statistical analysis | Low risk of bias |
| George 2022 | Low risk of bias | Moderate risk of bias: final response rate of only 15.2%, however, information is given about the people who weren’t included | Low risk of bias | | Low risk of bias | Moderate risk of bias: no mentioning of confounders in analysis | Low risk of bias |
| Hardy 2022 | Low risk of bias | Low risk of bias | Low risk of bias | | Low risk of bias | Moderate risk of bias: confounders are not mentioned but could be of importance when measuring patient reported outcomes | Low risk of bias |
| Hofstede 2018 | Moderate risk of bias: multiple cohorts from different hospitals are used and there is no clear description of the inclusion and exclusion criteria each hospital used. | Moderate risk of bias: no information is given about the process of retrieving information in the different cohorts | Low risk of bias | | Low risk of bias | Moderate risk of bias: possible confounders are not mentioned | Low risk of bias |
| Lu 2021 | Low risk of bias | Moderate risk of bias: it is stated that 5% of the study population was lost to follow-up, however nothing is said about the characteristics of this particular group and the possible effect on the outcome | Low risk of bias | | Low risk of bias | Low risk of bias | Low risk of bias |
| Omran 2024 | Low risk of bias | Moderate risk of bias. 18% of THA patients was finally eligible. However they did address attrition and selection bias by categorizing the patients. | Low risk of bias | | Low risk of bias. | Moderate risk of bias. They attempted to control for confounders by adjusting for various patient characteristic, however other factors like the quality of rehabilitation or socio economic status may have been left out. | Low risk of bias |
| Paredes 2025 | Low risk | Low risk | Low risk | | Low risk | Moderate risk: confounding was addressed in the models however not all confounders such as comorbidities en psychological factors were included | Low risk |
| Singh 2010 | Low risk of bias/Moderate risk of bias: no exclusion criteria are mentioned | Moderate risk of bias: response rate at 5yrs was 52% which is only half of the study population. There is no information on the patient characteristics of this group and the effect on the outcome | Low risk of bias | | Low risk of bias | Low risk of bias | Low risk of bias |
| Singh 2013 | Low/Moderate risk of bias: no exclusion criteria are mentioned | Moderate risk of bias: no information is given on people lost on follow up and outcome and prognostic outcome information of this study population | Low risk of bias | | Low risk of bias | Low risk of bias | Low risk of bias |
| Tang 2023 | Low risk of bias | Moderate risk of bias: of the initial 300 patients 43 were excluded with appropriate reason, however it could still influence the results. It is not clear how many of these 43 were THA patients. | Low risk of bias | | Low risk of bias | Moderate/high risk of bias: confounding for perioperative factors (medication, rehabilitation) and postoperative factors (opioid use, medication) was not accounted for | Low risk of bias |
| Ueki 2025 | Low/Moderate risk: no non-responder analysis | Low risk | Low risk | | Low/moderate risk: persistent pain defined as NRS but no functional impact is measured | Moderate/ High risk: confounders like age, sex, comorbidities and psychological factors were not included | Low risk |
| Wylde 2015 | Low risk of bias | Moderate risk of bias: no information on patients who were lost on follow up after first recruitment | Low risk of bias | | Low risk of bias | Low risk of bias | Low risk of bias |
